# Hypoxia tolerance of intertidal triplefin fish is associated with low critical oxygen tension and high phosphorylating capacity in brain mitochondria

**DOI:** 10.1038/s41598-025-30078-2

**Published:** 2026-01-16

**Authors:** Jules B. L. Devaux, Tristan J. McArley, Neill Herbert, Anthony J. R. Hickey

**Affiliations:** 1 School of Biological Sciences, The University of Auckland, Auckland, 1142 New Zealand; 2 Institute of Marine Science, The University of Auckland, Auckland, 1142 New Zealand

**Keywords:** Respirometry, Pcrit, Physiology, Cytochrome C oxidase, Loss of equilibrium, Oxidative phosphorylation, Mitochondria, Energy metabolism, Ecophysiology

## Abstract

At the terminus of the O_2_ cascade, mitochondria play an important role in O_2_ utilisation and energy conservation, with adaptive modifications occasionally shared among hypoxia-tolerant species. Here, we sought to determine whether mitochondrial adaptations in brain tissue explain the hypoxia tolerance of New Zealand triplefin fishes (*Tripterygiidae*). We compared two intertidal species (*Bellapiscis medius* and *Forsterygion lapillum*), both likely adapted to hypoxia-reoxygenation exposures, and two subtidal species (*F. varium* and *F. malcomi*), which inhabit normoxic waters. To assess hypoxia tolerance, we determined loss of equilibrium (LOE) during hypoxia exposure and measured the critical O_2_ tension (P_crit_). Intertidal species displayed superior hypoxia tolerance as assessed by LOE and also had lower P_crit_ (LOE versus P_crit_ R^2^ = 0.99). High-resolution respirometry was used to measure mitochondrial respiration in homogenate and permeabilised fragments of brain. While a weak relationship was apparent between mitochondrial O_2_ binding affinity (mP_50_) and LOE, maximum phosphorylating O_2_ consumption (OxPhos) and O_2_ catalytic rates were strongly correlated with hypoxia tolerance. Although cytochrome-*c*-oxidase activity was highest in the most hypoxia-tolerant species *B. medius*, it was only weakly correlated with hypoxia tolerance across species. Notably, the high OxPhos capacity of intertidal species was not associated with higher whole animal resting O_2_ consumption, suggesting intertidal species maintain high capacity for ATP production without incurring increased basal energetic costs. While somewhat paradoxical, the low P_crit_/high OxPhos strategy of intertidal species may provide flexibility in the dynamic intertidal environment where short, severe periods of hypoxia are interspersed with high energy demand environmental conditions, including acute warming.

**Supplementary Information:**

The online version contains supplementary material available at 10.1038/s41598-025-30078-2.

## Introduction

Adaptations to hypoxia come in many forms and occur at all levels of biological organisation, from behavioural adjustments to transcriptomic alterations^1^. Ultimately, to benefit hypoxia tolerance, these adaptations must contribute to the ability of an organism to maintain energy balance under conditions where ATP production via efficient oxidative phosphorylation is compromised^2^. At the terminus of the O_2_ cascade, mitochondria produce ATP via pathways that oxidise carbon sources and reduce O_2_ to water^3^. Multiple adaptations of mitochondrial structure and function have been identified in hypoxia-tolerant organisms^4^; however, these vary across hypoxia and anoxia tolerant species^5–7^. In particular, mitochondrial adaptations in the brain, which is often highly sensitive to O_2_ deprivation^8^, appear to play a key role in hypoxia tolerance^9–11^. For instance, to conserve ATP, brain mitochondria of the anoxia-tolerant turtle (*Trachemys scripta*) appear to remodel, partially uncouple and decrease F_0_-F_1_-ATPase activity during anoxia^12,13^. Additionally, in the epaulette shark (*Hemiscyllium ocellatum*), the electron transport chain in brain mitochondria remodels in anoxia with a decrease in complex II mediated O_2_ consumption, and this may protect against oxidative stress during reperfusion^14^. Brain mitochondria of intertidal sculpin species (*Cottidae* family) display greater O_2_ affinities, which is partially explained by cytochrome *c* oxidase (CCO) isoforms having greater O_2_ binding properties^15^. It is anticipated that both higher mitochondrial O_2_ binding affinity and higher mitochondrial O_2_ consumption would facilitate improved O_2_ extraction^16^ from the blood to brain tissue, thereby allowing intertidal fish to better utilize available O_2_ during periodic hypoxia in rock pools.

The New-Zealand triplefin fish (Family *Tripterygiidae*) group consists of 26 endemic species of which most occupy stable, normoxic subtidal habitats^17^. Three species, however, are known to inhabit intertidal rock pools^18^, which can become severely hypoxic during night-time low tides^19–21^. While not linked to hypoxia, there is some evidence of selective pressure on mitochondrial genes within rock pool species relative to subtidal species^17^. Moreover, heart^22^ and brain^23^ mitochondria of the exclusively intertidal rock pool species *Bellapiscis medius* had greater respiratory efficiencies and stabilities than two strictly subtidal triplefin species when exposed to elevated temperatures. Relative to the two subtidal triplefins, brain mitochondria of *B. medius* also showed adaptations that enhance ATP production under acidifying conditions, which are prevalent in hypoxic brains^24^. This suggests that mitochondrial function may differ among triplefin species challenged with hypoxia. The present study includes four triplefin species, which occupy a range of habitats varying in terms of the likelihood of environmental hypoxia exposure. *Bellapiscis medius* is an intertidal specialist, which exclusively inhabits rockpools that can routinely decrease to less than 20% air saturated O_2_ during night-time low tides^19,25^. *Fosterygion lapillum* occupies lower intertidal rock pools and shallow subtidal habitats, and *F. varium* and *F. malcomi* are exclusively subtidal species occupying rocky reef habitats to depths of ~ 35 m^19,25^. We have previously shown that the intertidal species *B. medius* and *F. lapillum* have a lower critical O_2_ tension (P_crit_) than their subtidal counterparts *F. varium* and *F. malcolmi*^25^. This suggests intertidal triplefins have a superior ability to meet O_2_ demand associated with standard metabolic rate (SMR; an estimate of basal metabolic rate) in hypoxia and indicates they are also likely to be more hypoxia tolerant. In the present investigation, time to loss of equilibrium (LOE) during severe hypoxia exposure was measured alongside P_crit_ to establish differences in absolute hypoxia tolerance among the intertidal and subtidal species.

Therefore, the aim of this study was to determine whether adaptations in mitochondrial oxygen consumption contribute to variation in hypoxia tolerance among intertidal and subtidal triplefins. Firstly, we established differences in whole animal hypoxia tolerance among intertidal and subtidal species at 20 °C. We predicted that relative to subtidal species (*F. varium* and *F. malcolmi*), intertidal species (*B. medius* and *F. lapillum*) would have lower P_crit_, as has been previously established at 18 °C^25^, and also be more hypoxia tolerant as assessed by time to LOE. We then measured O_2_ consumption in vitro in brain homogenate and permeabilised brain and assessed the mitochondrial affinity to O_2_ (mP_50_) and O_2_ catalytic efficiencies (K_cat,app_) using high resolution respirometry at the same temperature. We chose brain as this excitable tissue is acutely sensitive to low O_2_^8^. Brain homogenate preparations were used as these contain endogenous substrates and allows for a greater O_2_ diffusion, likely more representative mitochondrial environment in situ^26,27^, and to some extent, in vivo. However, O_2_ and substrate diffusion barriers may remain in homogenate preparations, and tissue permeabilisation may be required to address maximal mitochondrial respiration. Therefore, measuring O_2_ consumption using both preparations provides complementary information regarding state-dependent mitochondrial respiration, including in the context of declining O_2_ (20.5 kPa to anoxia). In addition, given that others reported that CCO could be a determinant of hypoxia tolerance in other intertidal fish species^15^, we measured CCO activity and derived inhibition-curves using sodium-azide to determine whether control from CCO differed among species. Thus, we predicted that the mitochondria and CCO of hypoxia-tolerant intertidal triplefins would display a greater affinity to O_2_ relative to subtidal species.

## Material and methods

### Experimental animals and housing

The animals used in this study were male and female adult specimens collected from sites on the Northeast coast of the Auckland region, between February and June. The rock pool specialist *B. medius* (*BM*) was caught from high intertidal pools (> 1 m) using hand nets, while the occasionally intertidal and shallow subtidal species *F. lapillum (FL)* was caught using minnow traps from nearshore subtidal sites (< 1 m). The deeper dwelling exclusively subtidal specimens *F. varium* (*FV*) and *F. malcomi* (*FM*) were caught with hand nets on Scuba dives at a depth of 10-15 m. The experiments carried out in this study were performed at two research facilities. The fish used in the LOE trials and mitochondrial assays were housed in a recirculated seawater facility at the University of Auckland’s School of Biological Sciences. These fish were held in 30 L tanks provided with a constant flow of recirculated seawater (20 ± 1°C, air saturated, 200 μm filtered, 35 ppt salinity). The fish used for whole animal respirometry were housed at the Leigh Marine Laboratory in 30 L flow-through seawater tanks (20 ± 0.5 °C, air saturated, 200 μm filtered, 35 ppt salinity). All fish were acclimated to laboratory conditions between 2–4 weeks prior to the start of experiments and were fed ad libitum on a mixture of shrimp, mussel and fish. Mass and length data for the fish used in each part of the study are found in Table 1. Food was withheld for a period of 48 h prior to the start of experiments. All capture, housing and experimental procedures are reported in accordance with ARRIVE guidelines, were performed under the approval of the University of Auckland Ethics Committee (Approval 001,551). All methods were performed in accordance with the relevant guidelines and regulations.

**Table 1:** Anatomical features of the four New Zealand triplefin fish species used in each part of the present study. Data presented as mean ± s.e.m, with sample sizes indicated in brackets in the left-hand column. Brain mass to body mass ratio was determined in 8 individuals of each species used in the mitochondrial function trials. Statistical differences among species were tested using one-way ANOVA. Significance was set at P < 0.05, and significant post-hoc differences between species are shown by uncommon superscript letters.

	Study (n)	Body length (mm)		Body weight (g)		Brain (mg.g^-1^)	
B. medius	P_crit_ (10)	64.80	± 1.37^a^	2.71	± 0.11^a^		
	P_LOE_ (9)	62.33	± 3.01^a^	3.01	± 0.37^ab^		
	Mito. (8)	54.36	± 4.81^a^	2.54	± 0.29^a^	0.73	± 0.04^a^
	Total (27)	60.50	± 2.94^a^	2.75	± 0.17^a^		
F. lapillum	P_crit_ (10)	62.90	± 2.47^a^	2.13	± 0.08^a^		
	P_LOE_ (9)	67.89	± 1.72^ab^	2.32	± 0.10^b^		
	Mito. (8)	68.57	± 1.41^b^	2.32	± 0.07^a^	0.72	± 0.02^a^
	Total (27)	66.45	± 1.41^a^	2.26	± 0.06^a^		
F. varium	P_crit_ (10)	80.90	± 2.04^b^	4.63	± 0.32^bc^		
	P_LOE_ (9)	64.11	± 2.00^a^	3.94	± 0.18^a^		
	Mito. (8)	65.00	± 2.80^bc^	4.01	± 0.21^b^	0.44	± 0.01^b^
	Total (27)	70.00	± 2.43^a^	4.19	± 0.19^b^		
F. malcomi	P_crit_ (10)	75.20	± 2.90^b^	4.37	± 0.59^c^		
	P_LOE_ (9)	83.00	± 3.54^b^	7.12	± 0.32^c^		
	Mito. (8)	80.64	± 2.83^c^	6.84	± 0.25^c^	0.34	± 0.01^c^
	Total (27)	79.61	± 2.08^b^	6.11	± 0.38^c^		

### Whole animal respirometry and determination of critical oxygen tension

**Fig. 1:**
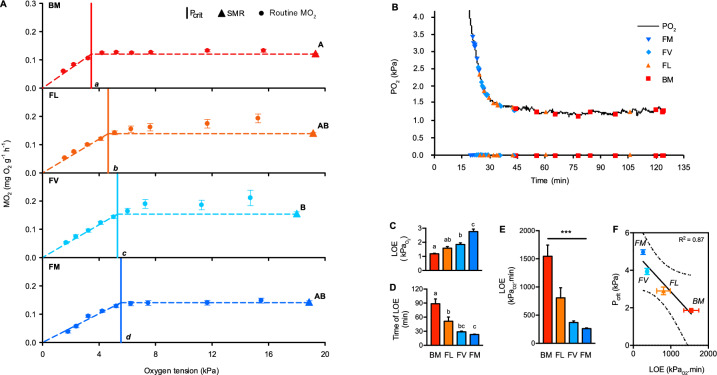
Critical O_2_ tension (P_crit_) and tolerance to hypoxia among intertidal and subtidal triplefin fish species. Bellapiscis medius (BM): exclusively intertidal rock pools; Forsterygiion lapillum (FL): intertidal rock pools and shallow subtidal; F. varium (FV): exclusively subtidal; F. malcomi (FM): exclusively subtidal. Data points show means ± s.e.m. N = 10 individuals for each species in panel A and N = 9 individuals for each species in panel C-E. (**A**) P_crit_ (vertical-coloured lines) was defined as the O_2_ partial pressure (PO_2_) at which mass-specific O_2_ consumption rate (measured by intermittent stop-flow respirometry) during stepwise hypoxia began to decline progressively below standard metabolic rate (SMR). (**B**) Profile showing the point of loss of equilibrium (LOE) for individuals of each species during a hypoxia exposure where PO_2_ was reduced from normoxia to < 1.5 kPa in ~ 30 min. (**C**) Partial pressure of O_2_ at LOE. (**D**) Time to LOE. (**E**) To account for the temporal aspect of hypoxia tolerance, total LOE was determined as the area above the curve of water O_2_ level versus time. (**F**) The relationship between P_crit_ and total LOE among species (dashed lines represent 95% confidence intervals). Differences in P_crit,_ SMR and LOE among species were tested with one-way ANOVA. Significance was set at P < 0.05, and significant post-hoc differences between species are shown by uncommon letters.

The P_crit_ of each species (N = 10; see Table 1 for mass and length) was determined at 20 °C using automated intermittent stop-flow respirometry^28^ to measure mass-specific O_2_ consumption ($\dot{M}$O_2_; mg O_2_ g ^-1^ h ^-1^). The design of the respirometers, general respirometry methods and procedure for $\dot{M}$O_2_ calculation are described in detail in McArley et al., (2018). P_crit_ was defined as the O_2_ tension where $\dot{M}$O_2_ under a progressive hypoxia exposure could no longer be maintained above standard metabolic rate (SMR; $\dot{M}$O_2_ in a rested, unfed animal)^29^. The protocol for P_crit_ determination began with an overnight period (~ 16 h) of respirometry where $\dot{M}$O_2_ was assessed repeatedly over 7–8 min cycles in undisturbed fish under normoxia. SMR was defined as the mean of the lowest 10% of $\dot{M}$O_2_ measurements made during the overnight period^30–33^, which likely corresponded to periods when fish were completely inactive as these species are benthic and tend to perch in a stationary position on the bottom of the respirometers. $\dot{M}$O_2_ measurements were then made at decreasing O_2_ tensions (~ 15.3, 11.6, 7.4, 6.3, 5.2, 4.2, 3.3, 2.3 and 1.6 kPa), with the required water O_2_ levels achieved by bubbling N_2_ into the seawater reservoir supplying respirometers. Three 7–8 min $\dot{M}$O_2_ measurements were made at 15.3, 11.6, 7.4, 6.3, 5.2 and 4.2 kPa, and one 7–8 min measurement at 3.3, 2.3 and 1.6 kPa. The entire progressive decline in O_2_ tension was completed in ~ 3 h, and the time of exposure to each O_2_ tension was the same for each species. To estimate P_crit_, SMR and routine $\dot{M}$O_2_ during progressive hypoxia were first mass corrected (see below) and then plotted against water PO_2_. A linear regression (forced through zero) was then established on $\dot{M}$O_2_ values that fell below SMR, and P_crit_ was calculated by dividing SMR by the slope of this regression line (i.e. the point where routine $\dot{M}$O_2_ under progressive hypoxia could no longer be maintained above SMR; see Fig. 1A) as per method of^34–37^.

To account for body mass differences between species (Table 1), $\dot{M}$O_2_ values were standardised to the mean body mass of all fish (3.5 g) from whole animal respirometry and P_crit_ assessments. Body mass correction of $\dot{M}$O_2_ values was carried out using the standard formula outlined in Schurmann and Steffensen^37^ and a mass scaling exponent of 0.8Clarke and Johnston^38^.

### Loss of equilibrium

Across three 40L tanks, three individuals of each species were placed into each tank (a total of 12 fish per tank) and left to recover for ~ 16 h in fully aerated (normoxic) seawater (see Table 1 for mass and length). Seawater PO_2_ in each tank was monitored using NeoFox-GT sensors (Ocean Optics^©^, Inc), and a clear plastic film was placed over the water surface to prevent the possibility of aerial surface respiration. After overnight recovery, hypoxia exposure was induced by bubbling N_2_ gas into the tanks holding the fish. Bubbling N_2_ continued until the tank PO_2_ reached the target water O_2_ level of ~ 1.4 kPa, which was achieved in approximately 30 min. We acknowledge that does not replicate the combined hypoxia–hypercapnia conditions of intertidal rock pools, where respiratory CO₂ accumulation may also influence oxygen transport and metabolism^39^. Once the target water O_2_ level was reached, N_2_ bubbling was adjusted manually to maintain a constant PO_2_ of ~ 1.4 kPa (Fig. 1.B). LOE was deemed to occur when a fish could no longer maintain an upright body position for a period of 10 s. In *FV* and *FM* and some *FL*, this occurred spontaneously, usually following a short burst of exercise, and occurred either before or soon after the target water PO_2_ was reached. In *BM* and the most hypoxia tolerant *FL*, however, individuals remained largely stationary on the tank bottom throughout the duration of the hypoxia exposure. To determine LOE in these individuals, the fish were periodically challenged by turning them over with a small stick and observing whether they could right themselves to an upright position within 10 s. To take into account the water O_2_ level of LOE and the length of time which hypoxia was survived, a composite measure of hypoxia tolerance was determined by the area above the curve of water O_2_ level versus time of hypoxia exposure. An O_2_ level of 21 kPa was set as the ceiling of the curve, and the analysis was performed using numpy.trapz module with default dx = 1 in Python 3.7. We referred to this composite measure as total LOE, and it was expressed in units of kPa_O2_.min.

### Tissue and mitochondrial respirometry

Fish were euthanized by rapid section of the spinal cord at the skull and the intact brain was dissected and then weighed ensuring any excess blood were removed. The “Iki Jime” method was chosen over the use of anesthetics as most affect mitochondrial functions^40^. Brains were then divided into two hemispheres for paired homogenate and permeabilisation assays. In experiments using homogenates, intact hemisphere was triturated gently by suction through a 10 ml syringe with decreasing gauge needles (16–25 gauge) prior to being introduced to the respirometry chamber, which occurred within 30 s of the initial brain dissection. In experiments using permeabilised samples, intact hemispheres were immediately placed in ice-cold biopsy buffer containing (in mM hereon, unless stated) 2.77 CaK_2_ EGTA, K_2_ 7.23 EGTA, 5.77 Na_2_ ATP, 6.56 MgCl_2_.6H_2_O, 20 taurine, 15 Na_2_-phosphocreatine, 20 imidazole, 0.5 DTT, 50 KMES, 50 sucrose, pH 7.22 at 20 °C^27^. Cellular permeabilisation was undertaken by the addition of 50 µg.ml^-1^ of freshly prepared saponin to plastic cell culture plates held on ice. The brains were then gently agitated in the culture plates for 30 min on ice, after which the permeabilised tissue was removed and washed three times for 10 min in ice-cold respiration medium (containing 0.5 EGTA, 3 MgCl_2_.6H_2_O, 60 K-lactobionate, 20 taurine, 10 KH_2_PO_4_, 2.5 HEPES, 30 MES, 160 sucrose, 1 g.l^-1^ BSA, pH 7.22 at 20 °C) prior to its addition to respirometry chambers. Fish were weighed and measured (see Table 1 for mass and length) after dissection and the proportion of brain mass relative to body mass was calculated.

Respiration was measured using Oroboros™ O2k high resolution respirometers (Innsbruck, Austria). The O_2_ electrode was calibrated from 0–20.46 kPa PO_2_ (0–100% air saturation, 262 µM dissolved O_2_ equivalent at 20ºC and 101 kPa) prior to respirometry assays. Brains (around ~ 2.5 mg homogenate or permeabilised) were introduced in the respirometry chambers containing 2 ml respiration medium calibrated prior experiment at 100% O_2_. After signal stabilisation (~ 10 min) and the measurement of the routine state in brain homogenates, saturating mitochondrial substrates (pyruvate, malate, glutamate and succinate) and ADP were added to maximise oxidative phosphorylation (OxPhos_PMGS_). The medium was then re-aerated fully and brains were left to deplete O_2_ and held in anoxia for ~ 5 min, after which re-oxygenation was performed. In chambers containing permeabilised brain, oligomycin (5 µM) was added to measure respiration attributed to proton leak (Leak), followed by carbonyl cyanide m-chlorophenyl hydrazone (0.5 µM titration steps until signal stabilisation) to measure the maximum O_2_ consumption capacity of the electron transport system (ETS) when uncoupled from OxPhos. In all assays, potassium-cyanide (1 mM) was added to measure non-mitochondrial O_2_ consumption, which was then subtracted from raw respiration data. Substrate-Uncoupler-Inhibitor-Titration protocols are detailed in the supplementary file (Table S.1).

###  Determination of mitochondrial affinity to O_2_

Respirometry data was recorded with DatLab (v7.1) software with the minimum smoothing to maximise resolution, especially at low PO_2_. Correction for time response of the electrode was also accounted for^41^. Above 2.05 kPa, an exponential moving average of 20 s (i.e. 10 recordings) was calculated in Excel to lower the signal to noise ratio. The mitochondrial affinity to O_2_ (mP_50_) was then determined as the PO_2_ at which the respiration rate is half of the maximum OxPhos rate. We also made an estimate of efficiency, which typically relates to kinetic parameters of purified enzymes. Given that mitochondrial respiration is a composite of pathways, we make a proxy for the classical k_cat_/K_M_ (turnover number/Michaelis constant) and present a measure of efficiency in the context JO_2Max_/mP_50_.

### Cytochrome c oxidase capacity and catalytic efficiency

The cytochrome c oxidase (CCO) capacity was assessed in two independent set of assays using two different methods (Table S.2). In permeabilised brain induced in OxPhos (saturated pyruvate, malate, glutamate, succinate and ADP), sodium azide was titrated to gradually inhibit CCO, until full CCO inhibition (final concentration of 12 mM). Inhibition dose response curves (Hill curves) were fitted with the least-squares method using GraphPad^©^ Prism. Using another set of assay, permeabilised brain was induced in uncoupled state, and electron feeding to CCO was inhibited by the addition of 2.5 µM antimycin A. Maximum CCO oxidation rates were then assessed by excess electron feeding with *N*,*N*,*N*’,*N*’-tetramethyl-p-phenylenediamine (TMPD, 0.5 mM) and additional ascorbate (2 mM) to measure TMPD auto-oxidation. Background chemical auto-oxidation was measured following additional potassium-cyanide (2 mM) and subtracted to CCO consumption rates. Net CCO rates were then normalised by the ETS capacity determined in the uncoupled state described in Sect. “Tissue and mitochondrial respirometry”.

### Statistical analysis

**Fig. 2:**
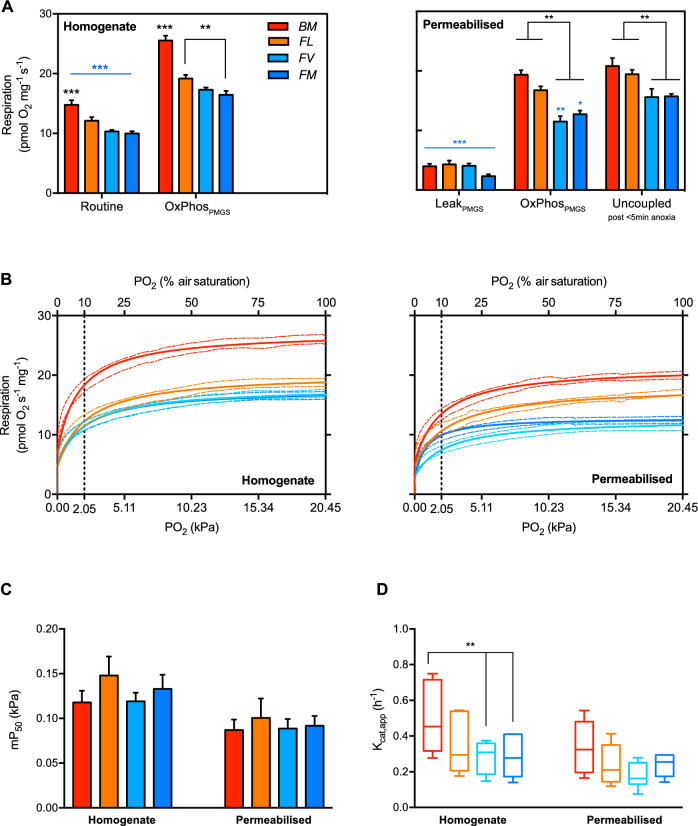
Oxygen kinetics at the cellular level. (**A**) Mitochondrial respiration was measured in brain homogenates (left) with no added substrates (i.e. only endogenous substrates present; “Routine”) and in the presence of saturated mitochondrial substrates pyruvate, malate and succinate (OxPhos_PMS_). Similarly, in permeabilised brain fragments (right), basal respiration attributed to proton leak (Leak_Omy_, measured in the presence of the ATP_F0-F1_ inhibitor oligomycin), OxPhos_PMS_ and maximum respiration when uncoupled from OxPhos_PMS_ with CCCP. (**B**) In air saturated medium and saturated respiratory substrates, oxygen consumption was measured in brain homogenate (left) and permeabilised brain (right), let to deplete O_2_ until anoxia. The dashed line at 2.05 kPa (10% air saturation) corresponds to the level below which the O_2_ tension is likely encountered intracellularly. (**C**) In both homogenate and permeabilised brain, all species displayed similar mitochondrial apparent affinity to O_2_ (mP_50_). Data expressed in kPa and extracted from the respiration curves in (B). (**D**) Brain homogenates of the most hypoxia tolerant species has a higher O_2_ turnover rate (JO_2Max_/mP_50_) than the two subtidal species. Species indicated as “BM”: B. medius, “FL”: F. lapillum, “FV”: F. varium and “FM”: F. malcomi. Data are mean of 8 and 7 (respectively homogenate and permeabilised) ± s.e.m. Statistical difference in black between species and in blue between states chosen at P < 0.05, 0.01 and 0.001 represented as *, ** and *** respectively.

**Fig. 3:**
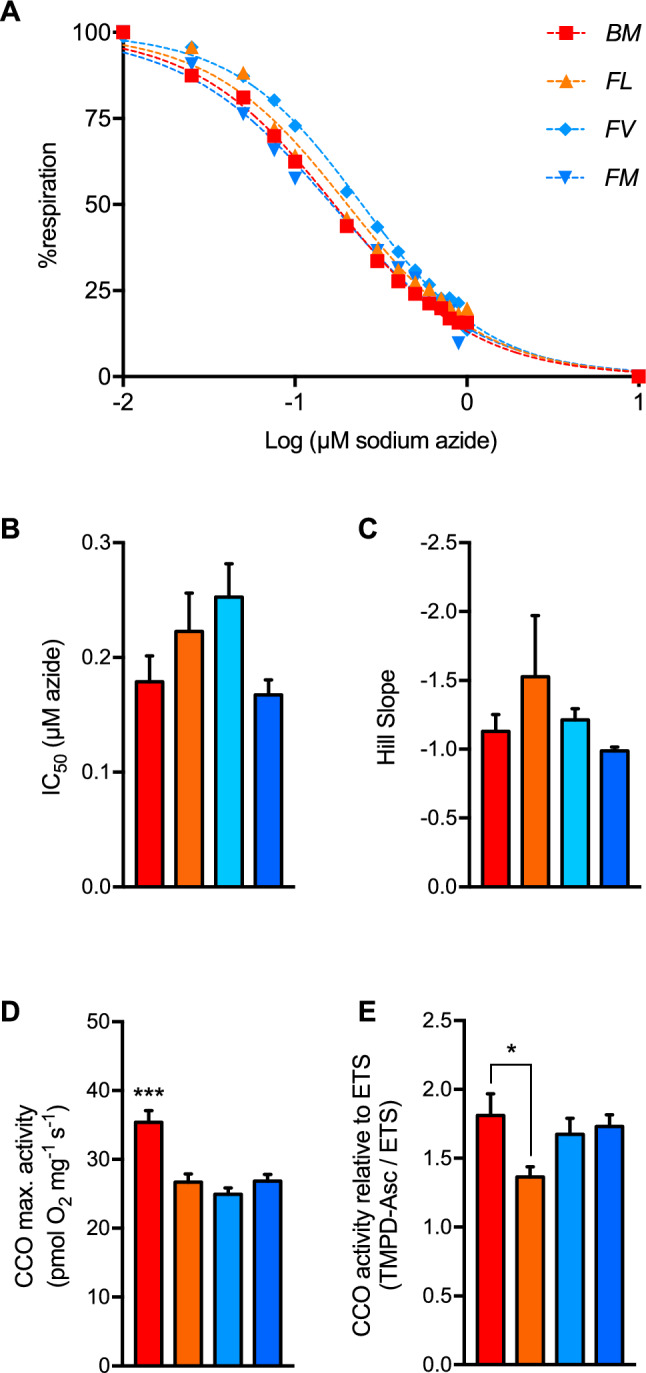
Cytochrome c oxidase properties in permeabilised brains of triplefin fishes. (**A**) Graded inhibition of cytochrome c oxidase was performed with the titration of sodium azide in situ. (**B**) Half inhibition (IC_50_) and (**C**) cooperativity (Hill slope) extracted from (A), with no apparent difference among fish species. (**D**) The maximum activity of the enzyme in situ was assessed with TMPD-ascorbate with correction for auto-oxidation and expressed in pmolO_2_.s^-1^.mg^-1^. (**E**) Affinity of an enzyme to its substrate, i.e. O_2_, is not independent of the enzyme concentration. Therefore, CCO activity was normalised by the mitochondrial electron transport system capacity (ETS). Intertidal fish species are “BM”: B. medius and “FL”: F. lapillum, and subtidal species are “FV”: F. varium and “FM”: F. varium. Data presented as mean of 6 individuals ± s.e.m. Statistical difference between species chosen at P < 0.05, 0.01 and 0.001 represented as *, ** and *** respectively.

**Fig. 4:**
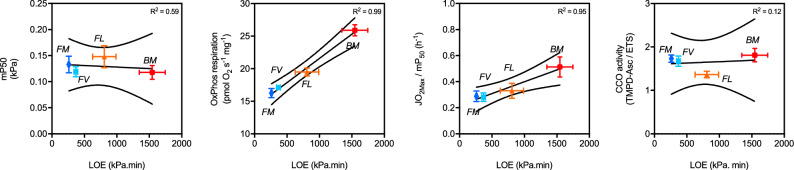
The paradox: Mitochondrial affinity to O_2_ does not correlate with hypoxia-tolerance but higher O_2_ extraction and consumption does. (**A**) mitochondrial affinity to O_2_ (mP50), (**B**) mitochondrial O_2_ consumption (homogenate OxPhos respiration), (**C**) O_2_ turnover rate and (**D**) maximal cytochrome c oxidase (CCO) activity correlation with loss of equilibrium (LOE) in the triplefin fish species (from most to least hypoxia-tolerant) “BM”: B. medius, “FL”: F. lapillum, “FV”: F. varium and “FM”: F. malcomi. Data extracted from previous figures using parameters from brain homogenates (A, B & C). Linear regression was performed using the least squares method and displayed as goodness of fit (full line) with confidence bands (dotted lines) chosen at 95%.

Statistical analyses were performed with GraphPad^©^ Prism 7, with significance set at P < 0.05. One-way ANOVA or two-way ANOVA followed by Tukey’s *post-hoc* tests were used to test for interactions and differences among and between species and/or parameters. For both mitochondrial respiration from 20.5 kPa to anoxia (Fig. 2.B) and CCO inhibition (Fig. 3.A) data, curves were fitted for each species to a three-parameters dose–response curve (Hill curve) using the least-squares method. Resulting fitted curves were then compared using the extra-sum of squares F-test to test for shared parameters, including mP_50_ (Fig. 2.C), IC50 and Hill slope (respectively Fig. 3.C and D). In Fig. 4, linear regression using least squares method was used to test for correlation between parameters of the mitochondrial function to LOE determined in 2.3.

## Results

### Hypoxia tolerance of triplefin fish species

SMR under normoxia was similar among species, except for *FV* having a slightly higher resting $\dot{M}$O_2_ than *BM* (0.15 ± 0.02 vs 0.11 ± 0.01 mgO_2_ g^-1^ h^-1^, respectively P < 0.05; Fig. 1.A). The intertidal specialist *BM* had a lower P_crit_ (3.45 kPa) than all other species (P < 0.05; Fig. 1.A), and the occasional intertidal occupant *FL* had a lower P_crit_ than the exclusively subtidal species *FV* and *FM* (P < 0.05; Fig. 1.A)*.* There was no difference in P_crit_ between the exclusively subtidal species *FV* and *FM*.

The two subtidal species could not match the lowest PO_2_ achieved by the two rock-pool species and lost equilibrium at ~ 2.8 ± 0.2 and 1.8 ± 0.1 kPa for *FM* and *FV*, respectively (Fig. 1.B). While some *FL* reached LOE around 2 kPa, the most tolerant *FL* maintained equilibrium for ~ 1.25 h at ~ 1.4 kPa (Fig. 1.B). *BM* tolerated severe hypoxia (~ 1.4 kPa) for an average time of 1.5 h before LOE. Both the O_2_ tension at LOE (Fig. 1.C) and the time duration to LOE (Fig. 1.D) showed *BM* and *FL* to have superior hypoxia tolerance than their subtidal counterparts. Indeed, when total LOE was expressed as the area above the curve from 0 min to time of LOE during hypoxia exposure, this revealed that the exclusively intertidal *BM* was more hypoxia tolerant than all other species and that the occasional intertidal occupant *FL* tended to be more hypoxia tolerant than the exclusively subtidal species (P < 0.05; Fig. 1.E). A strong linear correlation between P_crit_ and total LOE was verified among species (P < 0.01 ,R^2^ = 0.99, Fig. 1.F).

### Oxygen kinetics of brain tissues

Overall, O_2_ consumption was higher in the brain of *BM* relative to the subtidal species *FV* and *FM*, regardless of preparation or mitochondrial state (P < 0.01; Fig. 2.A). With the brain homogenate of all species, routine respiration was ~ 31% lower than OxPhos respiration (P < 0.01). In permeabilised brain without ADP, leak respiration rates were less than a third of OxPhos rates (P < 0.01) and only *FV* and *FM* showed an increase in O_2_ consumption with uncoupling (P < 0.05). OxPhos was however ~ 20% lower in brain homogenates of *FV* and *FM* relative to *BM* (P < 0.05).

To span the O_2_ levels the brain likely encounters in vivo, we assessed O_2_ consumption kinetics in vitro with brain homogenate and permeabilised brain in the OxPhos state (i.e. without substrate limitation). In brain homogenates, respiration was the highest in *BM* (P < 0.001), which retained almost two times that of the other species at PO_2_ < 2.05 kPa (Fig. 2.B). Respiration rates in *FL* was significantly higher than in *FV* and *FM* at PO_2_ > 2.05 kPa (P < 0.05), while at PO_2_ < 2.05 kPa, it was similar to the two subtidal species. Similarly, in permeabilised brain, respiration was more than 75% higher for the two intertidal species (P > 0.001;). In all species but *FL*, respiration rates were ~ 30% lower in permeabilised tissues than in homogenised tissues (P < 0.0001).

No difference in mP_50_ was found between the fish species with no correlation between maximum OxPhos respiration (Fig. 2.C). However, in brain homogenates the JO_2max_/mP_50_ was highest in the rock-pool *BM* relative to the intermediate and subtidal species (P < 0.01). This relationship was less apparent for permeabilised brain (Fig. 2.D).

### Cytochrome C oxidase

Dose response CCO inhibition by sodium azide was different among species (F_6,375_ = 7.16; P < 0.001; Fig. 3.A). Although, IC_50_ (Fig. 3.B) and Hill slopes (Fig. 3.C) extracted from the fitted curves were not significantly different. The *Forsterygion* genus had the lowest CCO activities (P < 0.001; Fig. 3.D), relative to that of the most hypoxia-tolerant *BM* (P < 0.05), with 10% more activity than the least hypoxia-tolerant *FM*.

### Correlation between mitochondrial function and hypoxia tolerance

At the subcellular level, mitochondrial O_2_ affinity (mP_50_) had a weak correlation with species hypoxia tolerance, here represented with total LOE (R^2^ = 0.51; Fig. 4.A). Paradoxically, higher homogenate O_2_ consumption rates in OxPhos state (Fig. 4.B) and greater mitochondrial O_2_ catalytic rates (Fig. 4.C) correlated strongly with hypoxia tolerance (R^2^ = 0.99 and 0.95, respectively). At a lower biological level, however, activity rates of CCO (the enzyme at the end of the O_2_ cascade) did not correlate linearly with total LOE (R^2^ = 0.12).

## Discussion

### Intertidal triplefins have superior hypoxia tolerance and lower P_crit_ than their subtidal counterparts

Here, we show intertidal triplefin species survive severe hypoxia for longer than their subtidal counterparts, which likely reflects the fact that the rock pools inhabited by these species can become severely hypoxic during night-time low tides^25^. We and others have previously demonstrated intertidal triplefin species have a lower P_crit_ than subtidal triplefin species and have speculated this should promote tolerance to hypoxia within this family of fish^22,25^. In the current study, the association between lower P_crit_ in intertidal species and improved hypoxia tolerance was confirmed by a strong inverse correlation between total LOE and newly measured P_crit_ among the four species examined. Time to LOE and P_crit_ were also correlated among intertidal and subtidal sculpin species^42^, which together with the current findings suggests that a relatively lower P_crit_ is likely important to the hypoxia survival strategy of intertidal fishes not undergoing metabolic depression. However, it is crucial to recognise that the interpretation of P_crit_ as a direct marker of hypoxia tolerance is increasingly being questioned. It is important to note that while P_crit_ is generally interpreted as the oxygen tension at which oxygen supply capacity is maximized, reflecting the upper limit of an organism’s ability to sustain aerobic metabolism (i.e., when \alpha is maximal), there is ongoing debate about its utility as a direct marker of hypoxia tolerance. Rather than a strict survival threshold, P_crit_ is increasingly viewed as an indicator of aerobic scope at a given PO_2_^43^. This nuanced understanding of P_crit_ emphasises its role in aerobic performance rather than solely as a measure of hypoxia tolerance, suggesting that the relationship between P_crit_ and hypoxia survival is more complex than previously assumed^43^. We also note that P_crit_ of intertidal triplefins were similar to these of “normoxic” species^44^, reinforcing the idea that absolute Pcrit values may not truly reflect the level of hypoxia tolerance of a species. In the absence of metabolic rate depression, which remains unobserved in triplefins, a lower P_crit_ would enable a greater proportion of energetic demands to be met through efficient mitochondrial ATP production as hypoxia intensifies. The hypoxia tolerant *BM* maintained an $\dot{M}$O_2_ equivalent to 51% of SMR at a PO_2_ of ~ 1.5 kPa, whereas the least hypoxia tolerant *FM* had an $\dot{M}$O_2_ equivalent to only 27% of SMR at the same PO_2._ The ability of *BM* to extract more O_2_ from severely hypoxic water is explained, at least partly, by the possession of gills with a high diffusive capacity for O_2_. Indeed, gill secondary lamellae are thin and numerous in this species^25^. Furthermore, relative to *FM*, *BM* has a higher blood O_2_ carrying capacity as indicated by higher haemoglobin concentration^25^. We also believe the typical behaviour of *BM* observed during LOE determination, where this species remains relatively inactive and stationary on the tank bottom, is likely to permit maintenance of a metabolic rate close to SMR at water PO_2_ below P_crit_. This behaviour contrasts starkly with that of the exclusively subtidal species, which become active, likely in an attempt to behaviourally avoid hypoxia, as water PO_2_ descends below P_crit_ and LOE is approached. Of course, high activity levels mean that metabolic rate will increase substantially beyond that associated with SMR and maintaining energy balance will become even more challenging. For subtidal species, hypoxia is escapable in nature and a behavioural avoidance strategy would be advantageous. However, hypoxia is inescapable in rock pools and also within the LOE trials we performed, so the quiescent low energy demand behaviour of *BM* is likely to convey a greater chance of surviving periods of severe hypoxia that develop in rock pools prior to replenishment of water O_2_ levels on the incoming tide. Quiescent behaviour in severe hypoxia has also been observed in other hypoxia tolerant intertidal fishes^45^. Lastly, we have previously demonstrated intertidal triplefins have higher white muscle and hepatic glycogen stores than subtidal species, which could also contribute to superior hypoxia tolerance through fuelling anaerobic respiration^25^. Large tissue glycogen stores and higher glycolytic enzyme activities in brain have also been demonstrated in intertidal sculpins^42^.

### Oxygen utilization is greater in brain mitochondria of intertidal species

Brain mitochondria of hypoxia-tolerant and hypoxia sensitive triplefin species had similar affinities to O_2_, with mP_50_ a little above 0.10 kPa**.** In comparison, the least hypoxia-tolerant sculpin displayed brain tissue mP_50_ of around 0.08 kPa, although this was measured 2ºC below our experimental temperature (i.e. 18ºC vs 20ºC)^15^ and higher temperatures decrease binding affinities^46^. Note that within sculpin species a strong correlation was observed between isolated mitochondria mP_50_ determined at 18ºC and P_crit_ measured at 12ºC^15^. In triplefin species, no such correlation was verified when measuring both parameter at 20ºC using brain homogenates or permeabilised tissue. We opted for determination of mP50 in homogenates and permeabilised brain tissue over isolated mitochondria, which is a common preparation method for measuring mitochondrial O_2_ affinities. We contend that mitochondrial isolation tends to select the more “robust” and “high-quality” mitochondrial sub-populations, and the disruption of mitochondrial membranes may lead to the loss of NAD(H_2_) pools, which could potentially restrict electron inputs to the electron transport chain. Additionally, low mitochondrial densities necessitate pooling of small triplefin brains for mitochondrial isolation^24^, significantly increasing animal sample size, which was not ethically justified.

Intracellularly, recent measurements of mitochondrial PO_2_ in vivo indicate levels of around 2.6–5 kPa^47,48^, levels at which brain homogenate mitochondria operated at ~ 70% of OxPhos for all fish species. Routine respiration rates, mediated by endogenous substrates and closely representative of in vivo conditions, were also ~ 70% that of OxPhos, yet routine respiration was measured under air saturated O_2_. This indicates that above 2 kPa, substrates, but not O_2,_ limit mitochondrial respiration in brain homogenates. Interestingly, subtidal species lost equilibrium around 2 kPa, which indicates that below such level, routine respiration may be affected by O_2_ levels, altering brain metabolism which subsequently compromises fish spatial awareness and reflexes upon acute exposure. Such PO_2_ is more than 25 times higher than mP_50_ measured here in vitro in both subtidal and intertidal fish, which indicates that all species studied here have marginal PO_2_ scope before reaching 50% of maximal mitochondrial respiration capacities.

In both homogenate and permeabilised brain, basal O_2_ consumption (Routine and Leak, respectively) and OxPhos were consistently greater in the intertidal hypoxia-tolerant species. As indicated by the greater JO_2max_/mP_50_ (a proxy for k_cat_/K_m_), intertidal species also have greater efficiency to bind and turnover O_2._ This at first appears paradoxical, given that O_2_ is limiting in hypoxia and that the mP_50_ of mitochondria are similar among species. However, elevation of O_2_ consumption can increase O_2_ gradients^16^, thereby enhancing O_2_ flow to mitochondria to better support ATP synthesis in hypoxia sensitive species. However, the elevated O_2_ consumption observed in brain tissue was not associated with a higher whole animal $\dot{M}$O_2_ in intertidal species. Thus, if the high basal O_2_ consumption and OxPhos observed in brain is common in other tissues of intertidal species, it does not come at the cost of a higher resting metabolic demand, which would likely impair hypoxia tolerance through increasing basal energetic demands. We have also observed that intertidal triplefins can achieve a higher maximum O_2_ consumption rate than their subtidal counterparts^25^, and this matches the high OxPhos observed in brain tissues of intertidal species here. Rather than high metabolic capacity being an adaptation for hypoxia tolerance per se, it might be that intertidal species retain the potential for high ATP production via OxPhos^24^, and this would meet the energy requirements of other high demand environmental conditions that occur in rock pools. For example, high aerobic capacity would be beneficial for meeting the energetic requirements of wave surge and for avoiding temperature-induced tissue O_2_ limitation (functional hypoxia) during acute warming events during daytime low tides. Moreover, thermal ramping events in rock pools tend to coincide with hyperoxia (O_2_ supersaturation)^2,25^, where the high OxPhos capacity of intertidal species could potentially act as a sink for excess O_2_ at the tissues and mitigate oxidative stress. This could also occur upon replenishment of rock pool water by the incoming tide, where a high OxPhos capacity could potentially mitigate oxidative stress associated with rapid hypoxia re-oxygenation of tissues^49^.

### Cytochrome c oxidase is not associated with hypoxia tolerance of triplefin fish

A greater mitochondrial O_2_ utilisation may reside from multiple adjustments. This includes a greater capacity to import and oxidise mitochondrial substrates, greater electron transport capacities of the ETS or a more tuned cytochrome *c* oxidase, the enzyme at the end of the O_2_ cascade. CCO subunit 4 has two paralogues in most vertebrates, including fish^50^, with CCO4-2 expression being elevated in response to hypoxia^51^, decreasing O_2_ affinity and consumption^52^. Notably, CCO inhibition curves were similar across species (Fig. 3**.A**), with similar apparent Km and Hill slopes, suggesting that triplefin species likely share similar forms of CCO with conserved O_2_ binding properties. In addition, no clear correlation between CCO and hypoxia tolerance was apparent. This again differs from sculpin species that showed a strong correlation between mP_50_ and CCO-O_2_ affinity^15^. Maximum CCO activity was similar to maximum OxPhos rates in *FL*, suggesting that unlike the other species, CCO reserve capacity is limited. This indicates O_2_ consumption and therefore ATP synthesis may be affected in *FL* if CCO activity were downregulated. As expected, however, the CCO capacity was higher than ETS capacity, and this suggests that CCO is not limiting and that regulation of O_2_ consumption in hypoxia-tolerant triplefin fish likely occurs at a higher biological level. It is also possible that species differences in hypoxia tolerance are partly influenced by variation in brain neuroglobin expression, which has been linked to neuronal survival under low oxygen conditions^53^.

## Conclusion

Here, we show that intertidal triplefin fish are more hypoxia tolerant than their subtidal counterparts. Indeed, the exclusively intertidal *BM* survived severe hypoxia (~ 1.4 kPa) for up to 2 h, whereas the exclusively subtidal *FM* could only tolerate brief periods of exposure to water O_2_ levels between ~ 2–3.5 kPa. Brain mitochondria in hypoxia-tolerant species displayed higher respiration rates than brain of hypoxia sensitive triplefins, even at PO_2_ where mitochondria function in vivo. High OxPhos capacity, while potentially aiding hypoxia tolerance through steepening O_2_ diffusion gradients between blood and tissues, may also provide reserve aerobic capacity to cope with other high energy demand environmental conditions that occur in the intertidal zone (e.g. acute warming). Although CCO, the enzyme at the end of the O_2_ cascade, was not correlated with hypoxia tolerance, it does not appear to limit O_2_ consumption in phosphorylating mitochondria. Despite similar mitochondrial affinity to O_2_ in brain tissue between hypoxia-tolerant and hypoxia sensitive triplefins, a greater capacity of intertidal species to access O_2_ in hypoxic environments was indicated by a lower P_crit_ at the whole animal level. Furthermore, intertidal species, despite the high OxPhos capacity identified in brain tissue, did not have increased resting $\dot{M}$O_2_. Combined with quiescent behaviour, this suggests that intertidal species are able to meet a greater proportion of their energetic demand through efficient mitochondrial ATP production, which would likely aid hypoxia tolerance in hypoxic rock pools inhabited by tripflefins, where severe hypoxia is of short duration and there is always some O_2_ available.

## Supplementary Information

Supplementary Information.

## Data Availability

The datasets generated and analysed during the current study are available in the Auckland University Figshare repository, https://auckland.figshare.com/articles/dataset/Hypoxia_tolerance_and_mitochondrial_function_in_intertidal_fish/29043959 (10.17608/k6.auckland.29043959).

## Abbreviations

CCO: Cytochrome C oxidase
ETS: Mitochondrial electron transport system
LOE: Loss of equilibrium
$\dot{M}$O_2_: Mass-specific O_2_ consumption
mP_50_: Oxygen tension at which mitochondrial respiration is half of its maximum
OxPhos: Oxidative respiration
P_crit_: Critical oxygen tension
PO_2_: Partial O_2_ pressure

## References

[1] Val, A. L., Silva, M. N. P. & Almeida-Val, V. M. F. Hypoxia adaptation in fish of the Amazon: A never-ending task. *S. Afr. J. Zool.* **33**(2), 107–114 (1998).

[2] Richards, J. G. Physiological, behavioral and biochemical adaptations of intertidal fishes to hypoxia. *J. Exp. Biol.* **214**(Pt 2), 191–199 (2011). 21177940 10.1242/jeb.047951

[3] Koch, L. G. & Britton, S. L. Aerobic metabolism underlies complexity and capacity. *J. Physiol.* **586**(1), 83–95 (2008). 17947307 10.1113/jphysiol.2007.144709 PMC2375572

[4] Pamenter, M. E. Mitochondria: A multimodal hub of hypoxia tolerance. *Can. J. Zool.* **92**(7), 569–589 (2014).

[5] Bickler, P. E. & Buck, L. T. Hypoxia tolerance in reptiles, amphibians, and fishes: Life with variable oxygen availability. *Annu. Rev. Physiol.* **69**, 145–170 (2007). 17037980 10.1146/annurev.physiol.69.031905.162529

[6] Gorr, T. A. et al. Hypoxia tolerance in animals: Biology and application. *Physiol. Biochem. Zool.* **83**(5), 733–752 (2010). 20565233 10.1086/648581

[7] Galli, G. L. & Richards, J. G. Mitochondria from anoxia-tolerant animals reveal common strategies to survive without oxygen. *J. Comp. Physiol. B* **184**(3), 285–302 (2014). 24504264 10.1007/s00360-014-0806-3

[8] Cervós-Navarro, J. & Diemer, N. H. Selective vulnerability in brain hypoxia. *Crit. Rev. Neurobiol.* **6**(3), 149–182 (1991). 1773451

[9] Larson, J. et al. No oxygen? No problem! Intrinsic brain tolerance to hypoxia in vertebrates. *J. Exp. Biol.* **217**(Pt 7), 1024–1039 (2014). 24671961 10.1242/jeb.085381 PMC3966918

[10] Nilsson, G. E. & Lutz, P. L. Anoxia tolerant brains. *J. Cereb. Blood Flow Metab.* **24**(5), 475–486 (2004). 15129179 10.1097/00004647-200405000-00001

[11] Del Rio, C. & Montaner, J. Hypoxia tolerant species: The wisdom of nature translated into targets for stroke therapy. *Int. J. Mol. Sci.* **22**(20), 11131 (2021). 34681788 10.3390/ijms222011131 PMC8537001

[12] Galli, G. L., Lau, G. Y. & Richards, J. G. Beating oxygen: Chronic anoxia exposure reduces mitochondrial F1FO-ATPase activity in turtle (Trachemys scripta) heart. *J. Exp. Biol.* **216**(Pt 17), 3283–3293 (2013). 23926310 10.1242/jeb.087155 PMC4074260

[13] Pamenter, M. E. et al. Mitochondrial responses to prolonged anoxia in brain of red-eared slider turtles. *Biol. Lett.* **12**(1), 20150797 (2016). 26763217 10.1098/rsbl.2015.0797 PMC4785919

[14] Devaux, J. B. L., Hickey, A. J. R. & Renshaw, G. M. C. Mitochondrial plasticity in the cerebellum of two anoxia-tolerant sharks: Contrasting responses to anoxia/reoxygenation. *J. Exp. Biol.* **222**, jeb191353 (2019). 30833461 10.1242/jeb.191353

[15] Lau, G. Y., Mandic, M. & Richards, J. G. Evolution of cytochrome c oxidase in hypoxia tolerant sculpins (Cottidae, Actinopterygii). *Mol. Biol. Evol.* **34**(9), 2153–2162 (2017). 28655155 10.1093/molbev/msx179

[16] Gnaiger, E. et al. Mitochondrial oxygen affinity, respiratory flux control and excess capacity of cytochrome c oxidase. *J. Exp. Biol.* **201**(Pt 8), 1129–1139 (1998). 9510525 10.1242/jeb.201.8.1129

[17] Hickey, A. J. et al. New Zealand triplefin fishes (family Tripterygiidae): Contrasting population structure and mtDNA diversity within a marine species flock. *Mol. Ecol.* **18**(4), 680–696 (2009). 19215584 10.1111/j.1365-294X.2008.04052.x

[18] Hickey, A. J. & Clements, K. D. Genome size evolution in New Zealand triplefin fishes. *J. Hered.* **96**(4), 356–362 (2005). 15858158 10.1093/jhered/esi061

[19] Hilton, Z., Wellenreuther, M. & Clements, K. D. Physiology underpins habitat partitioning in a sympatric sister-species pair of intertidal fishes. *Funct. Ecol.* **22**(6), 1108–1117 (2008).

[20] Hilton, Z., *Physiological adaptation in the radiation of New Zealand triplefin fishes (Family Tripterygiidae)*, in *School of Biological Sciences*, University of Auckland. 2010

[21] McArley, T. J., Hickey, A. J. R. & Herbert, N. A. Hyperoxia increases maximum oxygen consumption and aerobic scope of intertidal fish facing acutely high temperatures. *J. Exp. Biol.* **221**, jeb189993 (2018). 30254026 10.1242/jeb.189993

[22] Hilton, Z., Clements, K. D. & Hickey, A. J. Temperature sensitivity of cardiac mitochondria in intertidal and subtidal triplefin fishes. *J. Comp. Physiol. B* **180**(7), 979–990 (2010). 20461387 10.1007/s00360-010-0477-7

[23] Willis, J. R., Hickey, A. J. & Devaux, J. B. Thermally tolerant intertidal triplefin fish (Tripterygiidae) sustain ATP dynamics better than subtidal species under acute heat stress. *Sci. Rep.* **11**(1), 1–10 (2021). 34040122 10.1038/s41598-021-90575-y PMC8155050

[24] Devaux, J. B. L. et al. Acidosis maintains the function of brain mitochondria in hypoxia-tolerant triplefin fish: A strategy to survive acute hypoxic exposure?. *Front. Physiol.* **9**, 1941 (2019). 30713504 10.3389/fphys.2018.01941 PMC6346031

[25] McArley, T. J. et al. Intertidal triplefin fishes have a lower critical oxygen tension (Pcrit), higher maximal aerobic capacity, and higher tissue glycogen stores than their subtidal counterparts. *J. Comp. Physiol. B* **189**(3–4), 399–411 (2019). 30941501 10.1007/s00360-019-01216-w

[26] Gnaiger, E., *Mitochondrial physiology.* Bioenergetics Communications, 2020(BEC2020.1).

[27] Gnaiger, E., et al. *Mitochondria in the Cold*, in *Life in the Cold: Eleventh International Hibernation Symposium*, G. Heldmaier and M. Klingenspor, Editors. Springer Berlin Heidelberg: Berlin, Heidelberg. 431–442 2000.

[28] Steffensen, J. F. Some errors in respirometry of aquatic breathers: How to avoid and correct for them. *Fish Physiol. Biochem.* **6**(1), 49–59 (1989). 24226899 10.1007/BF02995809

[29] Claireaux, G. & Chabot, D. Responses by fishes to environmental hypoxia: Integration through Fry’s concept of aerobic metabolic scope. *J. Fish Biol.* **88**(1), 232–251 (2016). 26768976 10.1111/jfb.12833

[30] Mandic, M., Todgham, A. E. & Richards, J. G. Mechanisms and evolution of hypoxia tolerance in fish. *Proc. Biol. Sci.* **276**(1657), 735–744 (2009). 18996831 10.1098/rspb.2008.1235 PMC2660936

[31] Khan, J. R. et al. Optimum temperatures for growth and feed conversion in cultured hapuku (Polyprion oxygeneios) — Is there a link to aerobic metabolic scope and final temperature preference?. *Aquaculture* **430**, 107–113 (2014).

[32] Norin, T., Malte, H. & Clark, T. D. Aerobic scope does not predict the performance of a tropical eurythermal fish at elevated temperatures. *J. Exp. Biol.* **217**(Pt 2), 244–251 (2014). 24115064 10.1242/jeb.089755

[33] McArley, T. J., Hickey, A. J. R. & Herbert, N. A. Chronic warm exposure impairs growth performance and reduces thermal safety margins in the common triplefin fish (Forsterygion lapillum). *J. Exp. Biol.* **220**(Pt 19), 3527–3535 (2017). 28760830 10.1242/jeb.162099

[34] Behrens, J. W. & Steffensen, J. F. The effect of hypoxia on behavioural and physiological aspects of lesser sandeel, Ammodytes tobianus (Linnaeus, 1785). *Mar. Biol.* **150**(6), 1365–1377 (2007).

[35] Cook, D. G. et al. Low-O(2) acclimation shifts the hypoxia avoidance behaviour of snapper (Pagrus auratus) with only subtle changes in aerobic and anaerobic function. *J. Exp. Biol.* **216**(Pt 3), 369–378 (2013). 23038727 10.1242/jeb.073023

[36] Cumming, H. & Herbert, N. A. Gill structural change in response to turbidity has no effect on the oxygen uptake of a juvenile sparid fish. *Conserv. Physiol.* **4**(1), cow033 (2016). 27766155 10.1093/conphys/cow033 PMC5069868

[37] Schurmann, H. & Steffensen, J. F. Effects of temperature, hypoxia and activity on the metabolism of juvenile Atlantic cod. *J. Fish Biol.* **50**(6), 1166–1180 (1997).

[38] Clarke, A. & Johnston, N. M. Scaling of metabolic rate with body mass and temperature in teleost fish. *J. Anim. Ecol.* **68**(5), 893–905 (1999).

[39] Heuer, R. M. & Grosell, M. Physiological impacts of elevated carbon dioxide and ocean acidification on fish. *Am. J. Physiol. Regul. Integr. Comp. Physiol.* **307**(9), R1061–R1084 (2014). 25163920 10.1152/ajpregu.00064.2014

[40] Kishikawa, J. I. et al. General anesthetics cause mitochondrial dysfunction and reduction of intracellular ATP levels. *PLoS ONE* **13**(1), e0190213 (2018). 29298324 10.1371/journal.pone.0190213 PMC5752027

[41] Gnaiger, E. Polarographic oxygen sensors, the oxygraph, and high-resolution respirometry to assess mitochondrial function. *Drug-Induced Mitochondrial Dysfunction* **327**, 352 (2008).

[42] Mandic, M., Speers-Roesch, B. & Richards, J. G. Hypoxia tolerance in sculpins is associated with high anaerobic enzyme activity in brain but not in liver or muscle. *Physiol. Biochem. Zool.* **86**(1), 92–105 (2013). 23303324 10.1086/667938

[43] Seibel, B. A. et al. Oxygen supply capacity breathes new life into critical oxygen partial pressure (Pcrit). *J Exp Biol.* **224**(8), jeb242210 (2021). 33692079 10.1242/jeb.242210

[44] Seibel, B. A. et al. Breathing new life into the critical oxygen partial pressure (Pcrit): A new definition, interpretation and method of determination. *BioRxiv*10.1101/2020.09.01.278440 (2020).

[45] Yoshiyama, R. M. et al. Differential propensities for aerial emergence in intertidal sculpins (Teleostei; Cottidae). *J. Exp. Mar. Biol. Ecol.* **191**(2), 195–207 (1995).

[46] Blair, D. F. et al. Spectroelectrochemical study of cytochrome c oxidase: pH and temperature dependences of the cytochrome potentials. Characterization of site-site interactions. *J. Biol. Chem.* **261**(25), 11524–11537 (1986). 3017934

[47] Mik, E. G. Special article: measuring mitochondrial oxygen tension: from basic principles to application in humans. *Anesth. Analg.* **117**(4), 834–846 (2013). 23592604 10.1213/ANE.0b013e31828f29da

[48] Mik, E. G. et al. In vivo mitochondrial oxygen tension measured by a delayed fluorescence lifetime technique. *Biophys. J.* **95**(8), 3977–3990 (2008). 18641065 10.1529/biophysj.107.126094 PMC2553111

[49] Starkov, A. A. The role of mitochondria in reactive oxygen species metabolism and signaling. *Ann. N. Y. Acad. Sci.* **1147**, 37–52 (2008). 19076429 10.1196/annals.1427.015 PMC2869479

[50] Porplycia, D. et al. Subfunctionalization of COX4 paralogs in fish. *Am. J. Physiol. Regul. Integr. Comp. Physiol.* **312**(5), R671–R680 (2017). 28148493 10.1152/ajpregu.00479.2016 PMC5451570

[51] Fukuda, R. et al. HIF-1 regulates cytochrome oxidase subunits to optimize efficiency of respiration in hypoxic cells. *Cell* **129**(1), 111–122 (2007). 17418790 10.1016/j.cell.2007.01.047

[52] Pajuelo Reguera, D. et al. Cytochrome c oxidase subunit 4 isoform exchange results in modulation of oxygen affinity. *Cells* **9**(2), 443 (2020). 32075102 10.3390/cells9020443 PMC7072730

[53] Sun, Y. et al. Neuroglobin is up-regulated by and protects neurons from hypoxic-ischemic injury. *Proc. Natl. Acad. Sci. U S A.* **98**(26), 15306–15311 (2001). 11742077 10.1073/pnas.251466698 PMC65025

