# Supplementary material for: Hypoxia tolerance of intertidal triplefin fish is associated with low critical oxygen tension and high phosphorylating capacity in brain mitochondria
Source: Sci Rep. 2026 Jan 16;16:2202. doi: 10.1038/s41598-025-30078-2 (PMC12816700; doi:10.1038/s41598-025-30078-2)
Supplement: Supplementary file 1 — Supplementary Information. [file 41598_2025_30078_MOESM1_ESM.docx]

**Table S.1. Substrate Uncoupler Inhibitor Titration protocols employed in Fig.2.**

Chemical preparation and concentration were employed as recommended by Oroboros™

|  | **Homogenate** | |  | **Permeabilised** | |
| --- | --- | --- | --- | --- | --- |
|  | **Chemical** | **Mitochondrial state** |  | **Chemical** | **Mitochondrial state** |
| Steps |  |  |  |  |  |
| 1 |  | Routine |  | Pyruvate  + Malate  + Glutamate  + Succinate | Leak_PMG_ |
| 2 | Pyruvate  + Malate  + Glutamate  + Succinate  + ADP | OxPhos_PMGS_ |  | ADP | OxPhos_PMGS_ |
| 3 | Run into anoxia | OxPhos_PMGS_ |  | Run into anoxia | OxPhos_PMGS PO2-dependent_ |
| 4 |  |  |  | Reoxygenation (5min post-anoxia) | OxPhos_PMGS (post-5min anoxia)_ |
| 5 |  |  |  | Oligomycin | Leak_Omy_ |
| 6 |  |  |  | Carbonyl cyanide m-chlorophenyl hydrazone | Uncoupled |
| 7 |  |  |  | Potassium-cyanide | Non-mitochondrial respiration |

**Table S.2. Substrate Uncoupler Inhibitor Titration protocols employed in Fig.3.**

|  | **Figure 3. A, B, C** | |  | **Figure 3. D&E** | |
| --- | --- | --- | --- | --- | --- |
| E | **Chemical** | **Mitochondrial state** |  | **Chemical** | **Mitochondrial state** |
| Steps |  |  |  |  |  |
| 1 | Pyruvate  + Malate  + Glutamate  + Succinate  + ADP | OxPhos_PMGS_ |  | Pyruvate  + Malate  + Glutamate  + Succinate | Leak_PMG_ |
| 2 | Sodium azide  titration | CCO - inhibition |  | ADP | OxPhos_PMGS_ |
| 3 |  |  |  | Carbonyl cyanide m-chlorophenyl hydrazone | Uncoupled |
| 4 |  |  |  | Antimycin-A | Non-mitochondrial respiration |
| 5 |  |  |  | TMPD + Ascorbate | CCO |
| 6 |  |  |  | Potassium-cyanide | TMPD auto-oxidation background |

**Figure S.1. No effect of 5min anoxia on brain mitochondrial integrity.**

Respiratory control ratios (RCR) were calculated from the SUIT protocol employed in Table S.1 on permeabilised tissue. RCR pre-anoxia were calculated as OxPhos_PMGS_ (step3)/ Leak_PMGS_ (step1), and RCR post-anoxia were calculated as OxPhos_PMGS (post 5min anoxia)_ (step4) / Leak_Omy_ (step5). The absence of effect of anoxia on RCR was tested using two-way repeated measures ANOVA (P > 0.1), and difference between species was tested using post-hoc Turkey test. Data presented as mean ± s.e.m. of 6 individual and significance displayed as APA style.
